# Ganoderic Acid A Attenuates Pathological Cardiac Hypertrophy by Attenuating Inflammatory Responses

**DOI:** 10.3390/cimb48050471

**Published:** 2026-05-01

**Authors:** Changlin Zhen, Yonghui Zhang, Hui Tan, Dan Liu, Xiuzhen He, Wansong Chen

**Affiliations:** 1 School of Basic Medical Sciences, Chongqing Three Gorges Medical College, Chongqing 404120, China; 2 Chongqing Key Laboratory of Development and Utilization of Genuine Medicinal Materials in Three Gorges Reservoir Area, Natural Drug Intervention in Aging Key Laboratory of Chongqing Education Commission of China, Chongqing 404120, China

**Keywords:** cardiac hypertrophy, Ganoderic acid A, heart function, inflammation, NF-κB

## Abstract

Pathological cardiac hypertrophy is an important risk factor for cardiovascular disease. Ganoderic acid A (GAA), the primary bioactive constituent of *Ganoderma lucidum* (*G. lucidum*), is known for its stable chemical properties and diverse biological activities. It has been shown to confer protection against myocardial ischemia–reperfusion injury in rat models, potentially through modulating inflammatory responses and inhibiting protein expression linked to both NF-κB and apoptosis pathways. Nevertheless, the role of GAA in cardiac hypertrophy has not yet been fully elucidated. Using transverse aortic constriction (TAC)-induced cardiac hypertrophy in mice, we analyzed the degree of hypertrophy using echocardiography and at the pathology and molecular levels. Our results demonstrate that GAA effectively attenuates Ang II-induced cardiomyocyte hypertrophy in vitro and reduces pressure overload-induced cardiac hypertrophy in vivo. Further investigation revealed that GAA exerts its anti-hypertrophic effects by downregulating the mRNA expression of hypertrophic and fibrotic markers and attenuating inflammatory responses, and that the protective effects of GAA may involve NF-κB signaling. This study provides valuable theoretical support for the potential therapeutic application of GAA in treating pathological myocardial hypertrophy and heart failure.

## 1. Introduction

Cardiovascular disease remains a leading global cause of mortality and morbidity, with cardiac hypertrophy playing a significant role in its pathogenesis [1]. Common risk factors for cardiac hypertrophy include chronic increases in ventricular afterload resulting from conditions such as hypertension and aortic stenosis [2]. Pharmacological intervention remains a mainstream approach for managing and preventing cardiac hypertrophy, owing to its relatively high patient compliance and cost-effectiveness. However, while existing medications can alleviate clinical symptoms, their efficacy in preventing disease progression or reducing overall incidence and mortality remains limited. Thus, investigating novel therapeutic agents derived from medicinal and dietary homologous sources may offer promising strategies for suppressing cardiac hypertrophy.

Cardiac hypertrophy, induced by mechanical stimuli from conditions such as pregnancy, exercise, or sustained pressure overload, is an adaptive mechanism and is hence classified as physiological or pathological based on its origin [3,4]. Physiological hypertrophy, commonly induced by pregnancy or prolonged exercise, is a reversible condition that does not lead to impaired cardiac function [5]. In contrast to its physiological counterpart, pathological cardiac hypertrophy involves fibrotic remodeling and leads to a progressive decline in cardiac function, ultimately resulting in an irreversible condition [6]. The progression of pathological hypertrophy involves cardiomyocyte enlargement due to sustained increases in workload, leading to thickening of the ventricular wall. Persistent pressure overload can ultimately cause cardiac decompensation, which is characterized by apoptotic cell death and fibrotic tissue remodeling, and often progresses to heart failure [7]. Although significant progress has been made in understanding the molecular and pathogenic mechanisms of cardiac hypertrophy, options for effectively preventing or reversing this condition are still limited. Chronic inflammation, characterized by elevated inflammatory cytokines and low-grade systemic immune activation under physiological or environmental stress, has been identified through extensive clinical and experimental studies as a significant risk factor for cardiovascular diseases [8]. Immune cell infiltration and cytokine production are strongly correlated with hypertension and related end-organ damage. In essential hypertension, sustained pressure overload promotes the recruitment of inflammatory cells and pro-inflammatory cytokines, contributing critically to myocardial hypertrophy and structural remodeling [9]. Although both preclinical and clinical evidence—including the cardio-protective effects observed with anti-inflammatory therapies—supports a tight link between chronic inflammation and cardiac hypertrophy, the precise signaling mechanisms through which inflammation modulates cardiovascular pathology remain poorly defined. Elucidating these pathways and their regulatory mechanisms is essential for developing early interventional, preventive, and therapeutic strategies for cardiovascular diseases.

*Ganoderma lucidum* (*G. lucidum*), a fungal species within the Polyporaceae family (Basidiomycota), has been employed in traditional medicinal practices for more than 2000 years, particularly throughout Asia. Its fruiting bodies, cultured mycelia, and spores contain a variety of bioactive compounds, including polysaccharides, triterpenoids, and proteins [10]. Ganoderic acid A (GAA), a major triterpenoid constituent, is known for its chemical stability and diverse biological activities [11]. Previous research suggests that GAA possesses notable anti-inflammatory properties and shows promise in the prevention and treatment of cardiovascular disorders. For instance, it has been reported to alleviate toe joint inflammation in a rat model of collagen-induced rheumatoid arthritis by modulating cytokine levels and reducing the arthritis index [12]. Further studies by Zheng et al. demonstrated that GAA suppresses the expression of inflammatory mediators—such as COX-2 and NO—stimulated by IL-1β [13]. Furthermore, GAA has demonstrated protective effects against myocardial ischemia–reperfusion injury in rat models, likely mediated through the attenuation of inflammatory reactions and suppression of key proteins involved in both NF-κB signaling and apoptotic pathways [14]. Despite these findings, the role of GAA in cardiac hypertrophy remains largely unexplored.

This study aims to evaluate the therapeutic potential of GAA against cardiac hypertrophy through integrated histopathological and molecular assessments in both in vivo and in vitro experimental models. Moreover, the molecular mechanisms through which GAA regulates cardiac hypertrophy are comprehensively examined.

## 2. Materials and Methods

### 2.1. Ethical Permission

All procedures involving animals were conducted in accordance with the ethical standards and approved protocols of the Animal Experimentation Ethics Committee at Chongqing Three Gorges Medical College (approval no. SXYZ-A-2304-0008) and international animal ethics guidance, with the utmost efforts taken to minimize animal suffering. Chongqing Three Gorges Medical College Animal Experiment Center provided special training in animal care and handling for research staff.

### 2.2. Transverse Aortic Constriction

Transverse aortic constriction (TAC) surgery was performed using a well-characterized protocol [15]. Mice were individually placed in an induction chamber and anesthetized by inhalation of 3% isoflurane delivered in 100% oxygen at a flow rate of 1–2 L/min. Briefly, anesthetized mice underwent ligation of the aorta using a suture tightly secured around the vessel. A restrictive band (26 G) was placed around the aortic arch between the innominate and the left common carotid arteries. The severity of contraction was assessed 7 days after surgery by measuring the maximal flow velocity over the ligature with an MS400 probe of 10 MHz using a continuous Doppler (Vevo 2100, Visual Sonics, Toronto, ON, Canada). Mice with a maximum flow velocity of around 3000 mm/s were included in the study. Sham-operated animals received identical surgical exposure without aortic constriction. After 4 weeks of ligation, the animals were euthanized and samples were collected. During TAC surgery, there was a mortality rate of 20%, leading to exclusion of these deceased mice from further analysis. The analyses were randomized and blinded (echo/histology).

### 2.3. Animals

Male C57BL/6J mice were obtained from Cyagen Biosciences (Guangzhou, China) and maintained in a specific-pathogen-free (SPF) environment with regulated temperature (20–26 °C) and humidity (40–70%) and a 12 h light/dark photoperiod. All mice were provided with free access to a standard laboratory diet and water, and were randomly assigned to experimental groups. Ganoderic acid A (GAA) was purchased from ESITEBiogle (Chengdu, China; purity ≥ 95% by UV). For in vitro experiments, GAA was dissolved in 0.9% saline with 1% dimethyl sulfoxide (DMSO, Solarbio, Cat: D8370, Beijing, China). For in vivo experiments, we added 5 mg of GAA to 1 mL of CMC-NA solution and mixed thoroughly to obtain a homogeneous suspension with a working concentration of 5 mg/mL. Dosages of GAA and for both in vivo and in vitro applications were determined based on the previous literature and preliminary experiments [16,17]. The mice were randomly assigned to the following experimental groups (*n* = 6): Sham; TAC; Sham + GAA: one day before sham surgery, mice received oral administration of GAA (50 mg/kg), and from the day after sham surgery, mice received daily oral administration of GAA (50 mg/kg) for four weeks; TAC + GAA: one day before TAC surgery, mice received oral administration of GAA (50 mg/kg), and from the day after TAC surgery, mice received daily oral administration of GAA (50 mg/kg) for four weeks; TAC + CUT129: one day before TAC surgery, the NF-κB agonist CUT129 (selleck, S2979) [18] was administered via intraperitoneal injection at dose of 1 mg/kg, and from the day after TAC surgery, CUT129 was administered via intraperitoneal injection at a daily dose of 1 mg/kg for four weeks; TAC + GAA + CUT129: one day before TAC surgery, mice received oral administration of GAA (50 mg/kg) and CUT129 was administered via intraperitoneal injection at dose of 1 mg/kg, and from the day after TAC surgery, mice received daily oral administration of GAA (50 mg/kg) and CUT129 administered via intraperitoneal injection at a daily dose of 1 mg/kg for four weeks. After 4 weeks, the animals were sacrificed and samples were collected. No animals died before meeting criteria for euthanasia.

### 2.4. Euthanasia Procedure

At the conclusion of the experiment, all subjects were euthanized humanely to enable sample acquisition. Each mouse was placed into an induction chamber and anesthetized via inhalation of 3% isoflurane mixed with 100% oxygen, delivered at 1–2 L/min. Depth of anesthesia was assessed by monitoring the loss of pedal withdrawal and corneal reflexes to verify complete unconsciousness. Following confirmation of deep anesthesia, euthanasia was performed by decapitation. When rapid tissue collection was required, decapitation was conducted immediately after anesthetic induction to prevent recovery of consciousness. All steps were carried out by qualified personnel adhering to institutional and national ethical standards for animal research. Post-euthanasia, target tissues and organs were promptly collected. No animals died before meeting criteria for euthanasia.

### 2.5. Cell Culture and Treatments

For neonatal rat ventricular myocyte (NRVM) cultures, rats under three days old were sacrificed and their hearts were removed and washed with PBS to remove blood. The heart tissue was then digested using a mixture of 0.8 mg/mL pancreatin (T8150, Solarbio, Beijing, China) and 0.625 mg/mL collagenase (BY06021, Shanghai boyun biotech co., ltd., Shanghai, China), and the primary cardiomyocytes were removed from fibroblasts by applying the method of differential adherent culture. Finally, DMEM with 4.5 g/L glucose containing 10% fetal bovine serum and 5-BrdU (HY-15910, MedChemExpress, Monmouth Junction, NJ, USA) was used to culture the myocardium cells, which were then placed in a humidified incubator with 5% CO_2_ at 37 °C. After forty-eight hours of culture, the cardiomyocytes showed regular pulsation, suggesting that they could be used for follow-up experiments. Angiotensin II (Ang II) was obtained from Sigma (A9525, St. Louis, MO, USA). We evaluated the effect of GAA on cardiac hypertrophy via using 1 μM Ang II in NRVMs for 48 h. GAA: The NRVMs were treated with GAA (20 µM) for 52 h. GAA + Ang II: The NRVMs were pre-treated with GAA (20 µM) for 4 h and then treated with AngII (1 µM) for 48 h. CUT129: The NRVMs were treated with CUT129 (10 µM) for 52 h. CUT129 + GAA + Ang II: The NRVMs were pre-treated with CUT129 (10 µM) and GAA (20 µM) for 4 h and then treated with AngII (1 µM) for 48 h.

### 2.6. Echocardiography

Mice were anesthetized and underwent cardiac ultrasound examination with a Vevo 3100 imaging system (Visual Sonics, Toronto, ON, Canada) after undergoing TAC or sham operations. Left ventricular functional indices, such as end-diastolic diameter (LVID;d), end-systolic diameter (LVID;s), ejection fraction (EF%), and fractional shortening (FS%), were assessed following the manufacturer’s recommended protocols.

### 2.7. Histology

The hearts were perfused with phosphate-buffered saline (PBS) and fixed using 4% paraformaldehyde. Cross-sectional areas of cardiomyocytes were measured based on hematoxylin and eosin (H&E)-stained sections according to standardized methods [19]. Fifty cardiomyocytes per heart were evaluated, and the average cross-sectional area was determined. The cells were observed with a microscope (Leica Wetzlar, Hesse, Germany) and we measured the area of cardiomyocytes with Image-Pro Plus 6.0 software. In the Image-Pro Plus 6.0 system, we first outlined a sufficient number of cardiomyocytes (≥50 cells), and scale calibration was performed according to the picture to convert the pixel unit to μm. Then, the circle tool was utilized to measure the areas of cardiomyocytes and analysis was blinded. Collagen deposition was assessed via Masson’s trichrome staining [20].

### 2.8. Immunofluorescence Microscopy

Immunofluorescence staining was conducted following established protocols [21]. Following drug treatment, cardiomyocytes were fixed with 4% formaldehyde, permeabilized with 0.2% Triton, and incubated with a primary antibody targeting α-actinin (Proteintech, 11313-2-AP; dilution 1:500, Wuhan, China) or anti-p65 (CST, 8242, dilution 1:100, Beverly, MA, USA) and subsequently with an AffiniPure donkey anti-rabbit IgG (H+L) fluorescent secondary antibody (Jackson ImmunoResearch; dilution 1:200, West Grove, PA, USA). The cells were observed with a fluorescence microscope (Leica Wetzlar, Hesse, Germany) and we measured the area of cardiomyocytes with Image-Pro Plus 6.0 software. In the Image-Pro Plus 6.0 system, we first outlined a sufficient number of cardiomyocytes (≥50 cells), and scale calibration was performed according to the picture to convert the pixel unit to μm. Then, the circle tool was utilized to measure the areas of cardiomyocytes and analysis was blinded.

### 2.9. Western Blotting

Whole-cell lysates were extracted from both cultured cells and mouse heart tissues using lysis buffer, and protein concentrations were quantified with a BCA assay kit. Western blotting was performed as previously described [22]. Briefly, samples were normalized to equal protein concentrations, and 10 μL of each lysate was separated by SDS-PAGE. Proteins were then electrotransferred to a polyvinylidene fluoride (PVDF) membrane. The membranes were blocked with 5% bovine serum albumin (BSA) to minimize non-specific interactions and subsequently incubated with primary antibodies at 4 °C overnight. This was followed by 1 h of incubation with a horseradish peroxidase (HRP)-conjugated secondary antibody (ZSGB-BIO, goat anti-rabbit ZB-2301, Beijing, China). After washing, immunoreactive bands were detected and quantified with a Bio-Rad ChemiDoc MP Chemiluminescence Imaging System. Primary antibodies included: p65 (Proteintech, 80979-1-RR, 1:1000), phospho-p65 (Proteintech, 82335-1-RR, 1:1000), IkBα (Proteintech, 10268-1-AP, 1:1000), phospho-IkBα (Proteintech, 82349-1-RR, 1:1000), and α-tubulin (Proteintech, 80762-1-RR, 1:5000). The BCA protein quantification kit was obtained from Beyotime (Shanghai, China).

### 2.10. Quantitative Real Time-PCR

Total RNA was extracted from murine cardiac tissue and cultured cardiomyocytes using Trizol reagent (Beyotime, Shanghai, China) in accordance with the manufacturer’s instructions. First-strand cDNA was synthesized with a cDNA synthesis kit (Yeasen Biotech, Shanghai, China). Quantitative PCR was performed on an ABI 9500 Real-Time PCR instrument (Applied Biosystems, Carlsbad, CA, USA) employing Hieff UNICON^®^ Universal Blue qPCR SYBR Green Master Mix (Yeasen, Shanghai, China). Gapdh was used as the internal control, and relative gene expression was analyzed via the 2^−ΔΔCt^ method. All primer sequences are listed in Table S1.

### 2.11. Immunohistochemical Staining

Paraffin-embedded tissue sections were incubated with the antibodies of CD45 (ab10558, Abcam, Cambridge, UK) and CD68 (ab125212, Abcam) at 4 °C overnight, followed by incubation with the horseradish peroxidase regent for 30 min at 37 °C. Then, these sections were stained with DAB. Sections were assessed using a fluorescence microscope (Leica Wetzlar, Hesse, Germany) and further analyzed with the Image-Pro Plus software (version 6.0).

### 2.12. Statistical Analyses

Data are expressed as mean ± SEM. Statistical comparisons over time were evaluated using repeated-measures ANOVA, while intergroup differences were analyzed by one-way ANOVA followed by Bonferroni post hoc tests. Comparisons involving two groups were performed using Student’s *t*-test. A *p*-value of less than 0.05 was considered statistically significant.

## 3. Results

### 3.1. GAA Protected Against Angiotensin II-Induced Cardiomyocyte Hypertrophy

**Figure 1:**
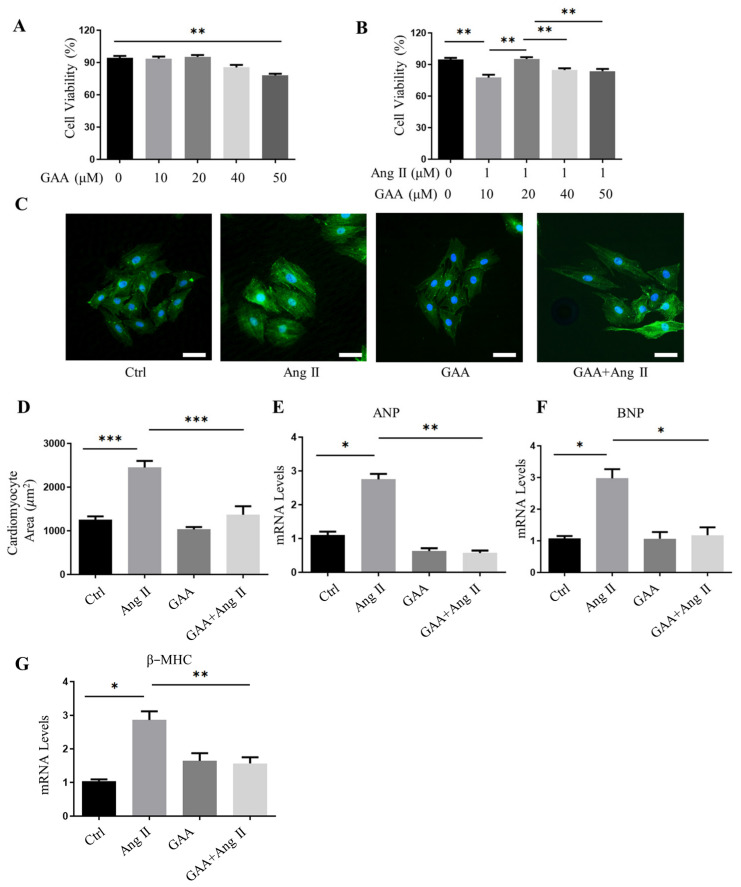
GAA alleviates hypertrophy in cardiomyocytes. (**A**) Cell viability detected in NRVMs treated with different concentrations of GAA (0, 10, 20, 40, 50 μM). (**B**) Cell viability detected in NRVMs treated with different concentrations of GAA as well as 1 μM Ang II. (**C**) Representative immunofluorescence images of NRVMs (green: α-actinin; blue: DAPI) treated with Ang II (1 µM) and GAA (20 μM). Original magnification ×200, scale bars = 50 µM. (**D**) Quantitative analysis of cell surface area (*n* ≥ 50 cells per group). (**E**–**G**) Real-time PCR analysis of mRNA expression levels for (**E**) ANP, (**F**) BNP, and (**G**) β-MHC in NRVMs under the indicated treatments (*n* = 3). All data are presented as the mean ± SEM; * *p* < 0.05, ** *p* < 0.01, *** *p* < 0.001.

The results of GAA cytotoxicity on rat ventricular myocytes (NRVMs) detected by Cell Counting Kit-8 (CCK-8) assay showed that high concentrations of GAA (40, 50 μM) resulted in a reduction in cell viability, and statistical analysis revealed that no distinct difference in cell viability was observed in other groups (Figure 1A). Based on the above results, GAA at concentrations of 10–20 μM had no cytotoxicity against NRVMs (Figure 1A). Compared with the vehicle group, the cell viability of angiotensin II (Ang II)-stimulated NRVMs was decreased, and treatment with 20–50 μM GAA restored the reduction (Figure 1B). Therefore, we selected the highest concentration that had no obvious effect on cell viability, 20 μM, as the in vitro administration dose. We initially investigated the protective effect of GAA against angiotensin II (Ang II)-induced hypertrophic responses in NRVMs. Exposure to Ang II for 48 h markedly increased the surface area of NRVMs, an effect that was significantly suppressed by concurrent GAA treatment (Figure 1C,D). The elevated expression of molecular markers such as atrial natriuretic peptide (ANP), B-type natriuretic peptide (BNP), and β-myosin heavy chain (β-MHC) serves as a hallmark of cardiac hypertrophy [23,24]. Elevated expression of ANP and BNP and a shift toward β-MHC are hallmarks of hypertrophic response [25]. Quantitative PCR analysis revealed that Ang II stimulation significantly upregulated the mRNA levels of ANP, BNP, and β-MHC. These increases were markedly suppressed by GAA treatment (Figure 1E–G). Together, these results suggest that GAA alleviates cardiomyocyte hypertrophy.

### 3.2. GAA Protected Against Pressure Overload-Induced Cardiac Hypertrophy In Vivo

**Figure 2:**
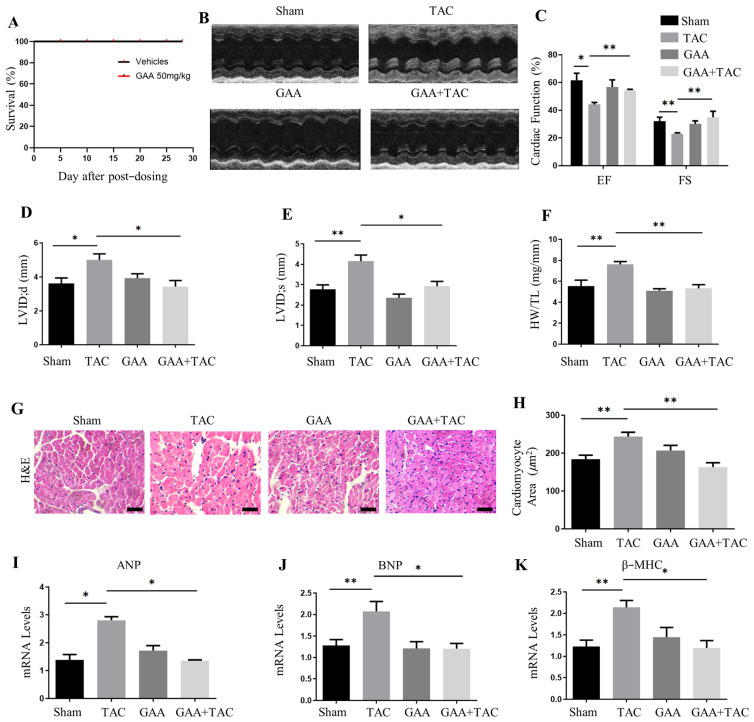
GAA protected against pressure overload-induced cardiac hypertrophy in vivo. (**A**) Survival curve of mice treated with GAA (50 mg/kg) and vehicle (*n* = 6). (**B**) Representative M-mode echocardiograms of the left ventricle. (**C**–**E**) Quantitative analysis of (**C**) EF% and FS%, (**D**) LVID;d, and (**E**) LVID;s from (**B**) (*n* = 6 per group). (**F**) Heart weight to tibia length (HW/TL) ratio post-surgery (*n* = 6). (**G**) Representative H&E-stained heart sections (scale bars: 100 µm). (**H**) Quantification of cardiomyocyte cross-sectional areas (*n* ≥ 50 cells). (**I**–**K**) mRNA expression levels of (**I**) ANP, (**J**) BNP, and (**K**) β-MHC, determined by RT-qPCR (*n* = 6). Data are mean ± SEM; * *p* < 0.05, ** *p* < 0.01.

**Figure 3:**
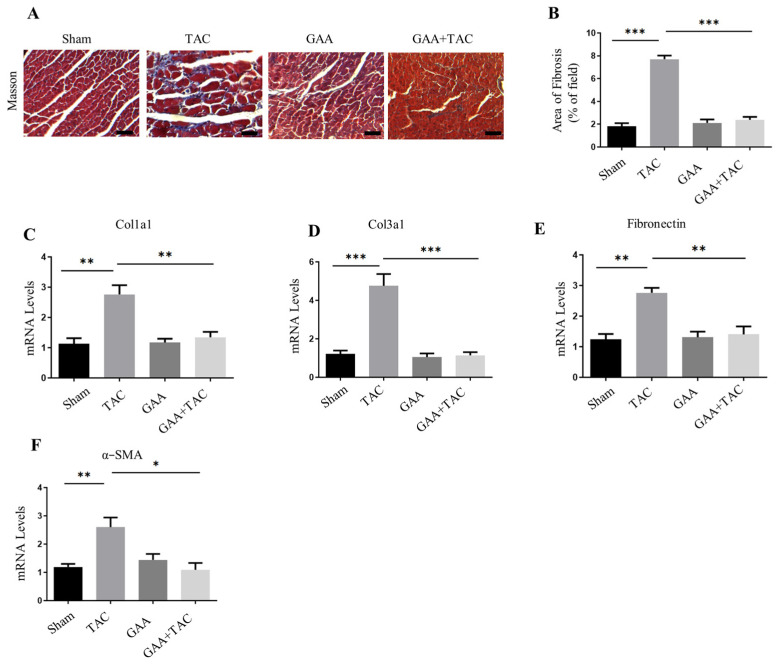
GAA alleviates cardiac fibrosis induced by TAC in vivo. (**A**) Representative Masson’s trichrome-stained heart sections depicting collagen deposition (blue). Scale bars: 100 µm. (**B**) Quantitative analysis of cardiac interstitial fibrosis (*n* = 6). (**C**–**F**) mRNA expression levels of fibrotic markers (**C**) Col1a1, (**D**) Col3a1, (**E**) fibronectin, and (**F**) α-SMA, as determined by RT-qPCR (*n* = 6). Data are presented as mean ± SEM; * *p* < 0.05, ** *p* < 0.01, *** *p* < 0.001.

Firstly, the result of the survival curve in mice treated with GAA shows that GAA (50 mg/kg) has no toxic effects on mice (Figure 2A). To assess the in vivo protective efficacy of GAA against cardiac hypertrophy, the transverse aortic constriction (TAC) model was employed. Post-operatively, wild-type (WT) mice were administered GAA via oral gavage for a four-week period. Echocardiographic assessment revealed that TAC-induced cardiac impairment in WT mice was characterized by a significant reduction in fractional shortening (FS%) and ejection fraction (EF%), coupled with an enlargement of left ventricular internal dimensions at both end-systole (LVID;s) and end-diastole (LVID;d) (Figure 2B–E). Administration of GAA attenuated these alterations, resulting in enhanced ejection fraction (EF%) and fractional shortening (FS%), as well as decreased left ventricular internal dimensions at end-systole (LVID;s) and end-diastole (LVID;d) (Figure 2B–E). Consistent with these findings, GAA administration markedly attenuated hypertrophic remodeling and lowered the heart weight to tibia length (HW/TL) ratio (Figure 2F). Histological evaluation using hematoxylin and eosin (H&E) staining demonstrated that GAA markedly diminished the TAC-elevated cardiomyocyte cross-sectional area (Figure 2G,H). Furthermore, GAA treatment led to the downregulation of hypertrophy-related gene expression, including of ANP, BNP and β-MHC, in myocardial tissue (Figure 2I–K). Masson’s trichrome staining indicated a significant reduction in cardiac fibrosis following GAA treatment relative to TAC control animals (Figure 3A,B). Correspondingly, qRT-PCR analysis indicated that GAA treatment inhibited the upregulation of fibrotic genes, including fibronectin, α-SMA, Col3a1 and Col1a1 (Figure 3C–F). Collectively, these findings indicate that GAA attenuates TAC-induced cardiac hypertrophy, fibrosis, and functional impairment.

### 3.3. GAA Attenuated Cardiac Hypertrophy by Attenuating Inflammatory Responses

**Figure 4:**
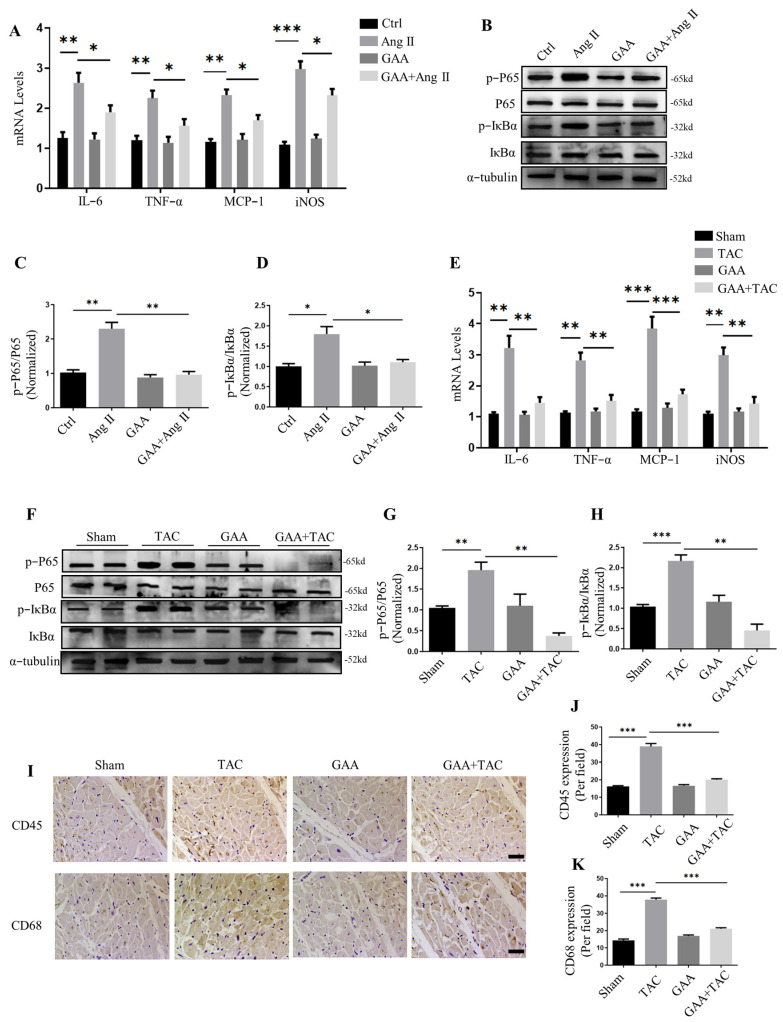
GAA attenuated inflammatory responses by inhibiting NF-κB pathway. (**A**) Real-time PCR analysis of mRNA expression levels for IL-6, TNF-α, MCP-1 and iNOS in NRVMs under the indicated treatments (*n* = 3). (**B**) Representative Western blots of p-IκBα and p-p65 levels in NRVMs treated with Ang II (1 µM) and GAA (20 μM). (**C**,**D**) Densitometric quantification of (**C**) p-p65 and (**D**) p-IκBα from (**B**) (*n* = 3). (**E**) mRNA expression levels of IL-6, TNF-α, MCP-1 and iNOS determined by RT-qPCR (*n* = 6). (**F**) Representative Western blots of p-IκBα and p-p65 in cardiac tissues from sham- or TAC-operated mice. (**G**,**H**) Quantification of (**G**) p-p65 and (**H**) p-IκBα from (**F**) (*n* = 6). (**I**) Immunohistochemical staining of CD45 and CD68 in the heart. Scale bars: 100 µm. (**J**,**K**) Quantification analysis of CD45- and CD68-positive areas (*n* = 6). Data are mean ± SEM; * *p* < 0.05, ** *p* < 0.01, *** *p* < 0.001.

Growing evidence suggests a strong association between persistent low-grade inflammation and the progression of cardiac hypertrophy. Chronic mechanical overload triggers immune cell infiltration and pro-inflammatory cytokine release, a key pathophysiological process that thereby fuels the development of myocardial hypertrophy and architectural remodeling [9]. Previous studies indicate that mechanical stress induces IκBα phosphorylation and proteolysis, a well-documented mechanism that culminates in NF-κB activation and nuclear translocation. This in turn upregulates the expression of inflammatory mediators including tumor necrosis factor-α (TNF-α), as well as hypertrophy-related genes such as ANP, thereby promoting the initiation and progression of cardiac hypertrophy [26]. Given that GAA has been reported to mitigate myocardial ischemia–reperfusion injury by attenuating inflammatory responses, we hypothesized that GAA may also attenuate inflammatory responses in the context of hypertrophy. In vitro, we examined inflammatory factors (e.g., IL-6, TNF-α, MCP-1, iNOS). Quantitative PCR analysis revealed that Ang II stimulation significantly upregulated the mRNA levels of IL-6, TNF-α, MCP-1 and iNOS (Figure 4A). These increases were markedly suppressed by GAA treatment (Figure 4A). Furthermore, GAA also inhibited the inflammation-related pathway–NF-kB signaling pathway. GAA treatment blocked the Ang II-induced elevation in phosphorylated IκBα and p65 levels (Figure 4B–D). Similarly, GAA treatment led to the downregulation of inflammatory factor expression, including IL-6, TNF-α, MCP-1 and iNOS in myocardial tissue (Figure 4E). In TAC-induced hypertrophic hearts, elevated levels of phospho-IκBα and phospho-p65 were observed, and these increases were markedly attenuated by GAA administration (Figure 4F–H). In addition, considering that CD45 and CD68 act as indicators of inflammation [27,28], immunohistochemistry (IHC) was conducted, and the results showed that GAA downregulated the amount of CD45- and CD68-positive cells in the TAC + GAA group compared to the TAC group (Figure 4I–K). These results indicate that GAA inhibited inflammatory cell infiltration. Collectively, these findings indicate that GAA attenuated cardiac hypertrophy by attenuating inflammatory responses.

### 3.4. Protective Effects of GAA Involve NF-κB Signaling

**Figure 5:**
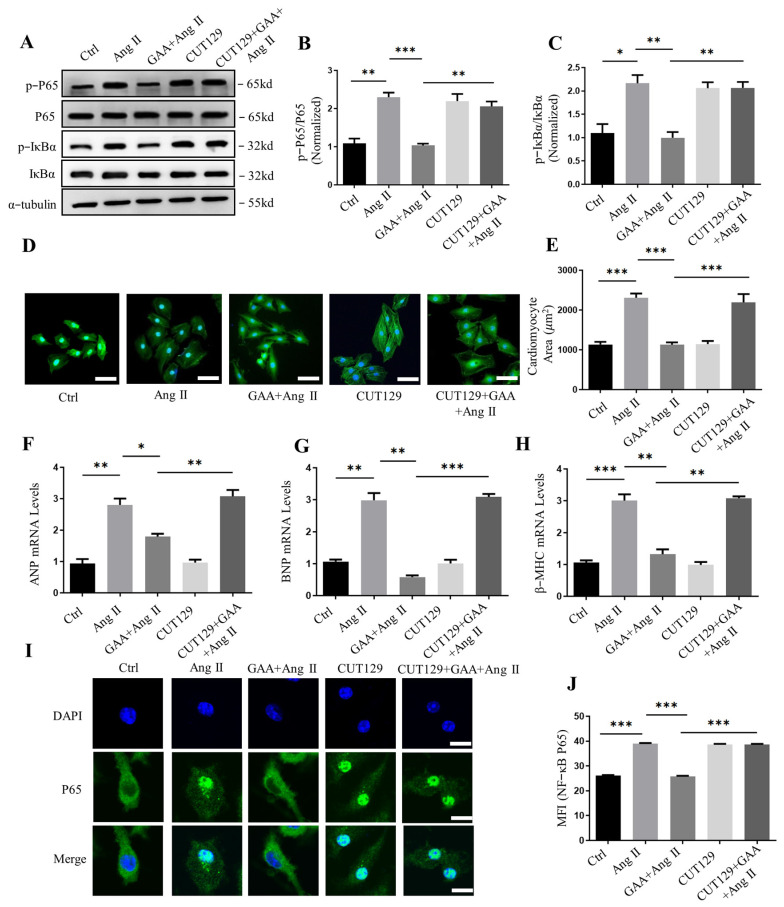
Protective effects of GAA involve the NF-κB signaling. (**A**) Representative Western blots of p-IκBα and p-p65 in NRVMs under the following treatments: Ang II (1 µM), GAA (20 μM), and CUT129 (10 µM, 4 h pre-treatment). (**B**,**C**) Respective quantifications of (**B**) p-p65 and (**C**) p-IκBα levels (*n* = 3). (**D**) Representative immunofluorescence images of cardiomyocytes (α-actinin: green; DAPI: blue) from the indicated groups. Scale bars: 50 µm. (**E**) Quantification of cell surface area (*n* ≥ 50 cells/group). (**F**–**H**) mRNA expression levels of hypertrophic markers (**F**) ANP, (**G**) BNP, and (**H**) β-MHC, measured by RT-qPCR (*n* = 6). (**I**) Images of NRVMs stained for p65. Original magnification ×200, scale bars = 50 µM. (**J**) Quantification of mean fluorescence intensity of analyzed NF-κB p65 nuclear translocation (*n* ≥ 50 cells/group). Data are mean ± SEM; * *p* < 0.05, ** *p* < 0.01, *** *p* < 0.001.

To determine whether NF-κB signaling is involved in the anti-hypertrophic effect of GAA, we treated cardiomyocytes with the NF-κB agonist CUT129 (10 μM). Treatment with CUT129 enhanced the phosphorylation levels of IκBα and p65, which counteracted the suppressive influence of GAA on NF-κB pathway activity (Figure 5A–C). Immunostaining for α-actinin showed that CUT129 abolished the attenuation of Ang II-induced cardiomyocyte hypertrophy by GAA (Figure 5D,E). Consistent with this, the downregulation of ANP, BNP, and β-MHC mediated by GAA was also negated upon CUT129 treatment (Figure 5F–H). Furthermore, we used NRVMs to detect the nuclear translocation of NF-κB p65. Compared to the control group, the nuclear translocation of NF-κB p65 was induced in NRVMs by Ang II stimulation (Figure 5I,J). However, the immunofluorescence intensity of cellular nuclear NF-κB p65 was reduced by GAA treatment (Figure 5I,J). Furthermore, the reduction in nuclear translocation of NF-κB p65 mediated by GAA was reversed upon CUT129 treatment (Figure 5I,J).

**Figure 6:**
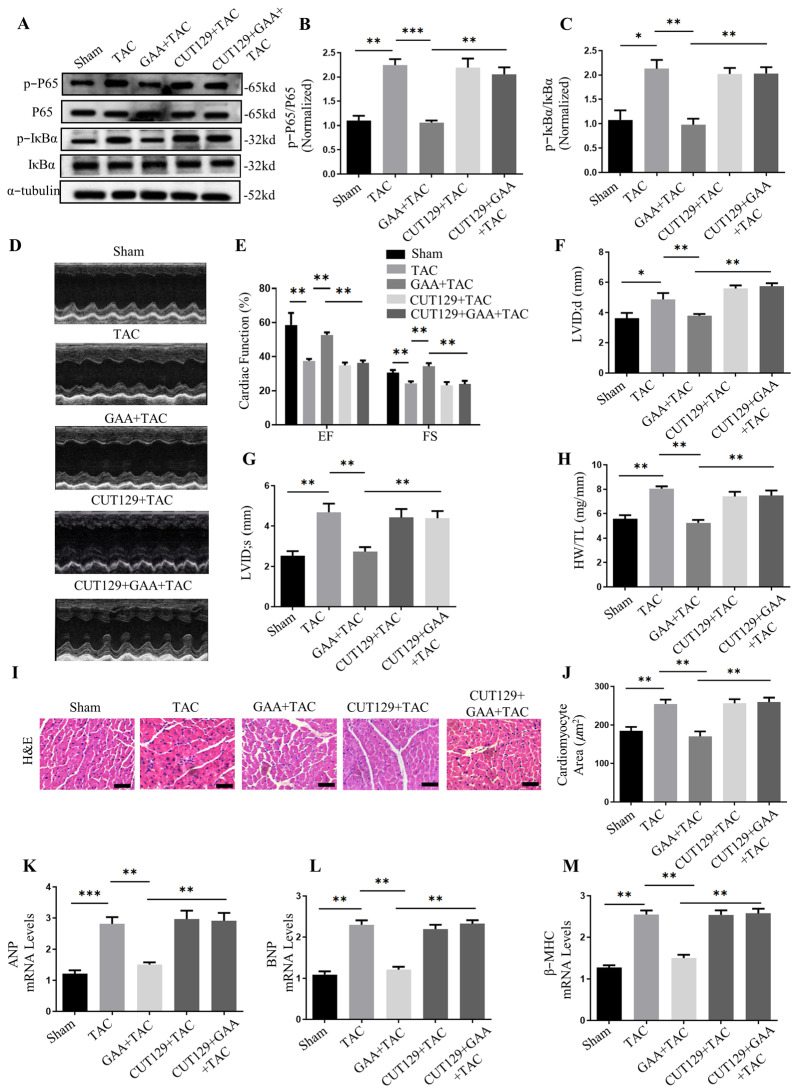
GAA increases cardiac function and ameliorates cardiac hypertrophy by inhibiting the NF-κB pathway. (**A**) Representative Western blots of p-IκBα and p-p65 in cardiac tissues from sham- or TAC-operated mice. (**B**,**C**) Quantification of (**B**) p-p65 and (**C**) p-IκBα from (**A**) (*n* = 6). (**D**) Representative M-mode echocardiograms of left ventricles. (**E**–**G**) Quantitative analysis of (**E**) EF% and FS%, (**F**) LVID;d, and (**G**) LVID;s (*n* = 6). (**H**) Heart HW/TL ratios post-surgery (*n* = 6). (**I**) Representative H&E-stained heart sections (scale bars: 100 µm). (**J**) Quantification of cardiomyocyte cross-sectional areas (*n* ≥ 50 cells). (**K**–**M**) mRNA levels of hypertrophic markers (**K**) ANP, (**L**) BNP, and (**M**) β-MHC by RT-qPCR (*n* = 6). Data are mean ± SEM; * *p* < 0.05, ** *p* < 0.01, *** *p* < 0.001.

**Figure 7:**
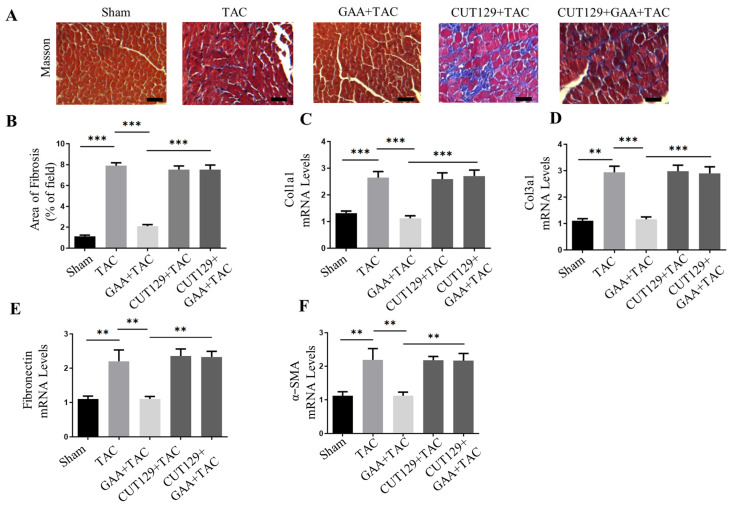
GAA attenuates cardiac fibrosis induced by TAC by inhibiting the NF-κB pathway in vivo. (**A**) Representative Masson’s trichrome-stained heart sections (scale bars: 100 µm). (**B**) Quantitative analysis of cardiac interstitial fibrosis (*n* = 6). (**C**–**F**) mRNA expression of fibrotic markers—(**C**) Col1a1, (**D**) Col3a1, (**E**) fibronectin, and (**F**) α-SMA—measured by RT-qPCR (*n* = 6). Data are mean ± SEM; ** *p* < 0.01, *** *p* < 0.001.

In vivo, mice were treated with the NF-κB agonist CUT129 (1 mg/kg/d). Treatment with CUT129 enhanced the phosphorylation levels of IκBα and p65 in mice heart issues, which counteracted the suppressive influence of GAA on NF-κB pathway activity (Figure 6A–C). Echocardiographic evaluation revealed that GAA alleviated cardiac hypertrophy and functional impairment induced by TAC, as evidenced by decreased LVID;s and LVID;d, accompanied by EF% and FS% (Figure 6D–G). However, co-administration of CUT129 counteracted these beneficial effects, resulting in increased ventricular dimensions and impaired systolic function (Figure 6D–G). Consistent with these findings, CUT129 treatment also reversed the reduction in HW/TL ratio conferred by GAA (Figure 6H). Furthermore, histological and molecular analyses revealed that CUT129 abrogated the anti-hypertrophic effects of GAA, leading to enlarged cardiomyocyte cross-sectional areas and elevated expression of hypertrophy markers (Figure 6I–M). Masson’s trichrome staining showed that the attenuation of cardiac fibrosis by GAA was diminished upon CUT129 administration (Figure 7A,B). Similarly, the suppressive effect of GAA on fibrosis-related genes was reversed by CUT129 treatment (Figure 7C–F). These results collectively indicated that NF-κB signaling is involved in the anti-hypertrophic effect of GAA. In summary, our findings indicate that GAA alleviates pressure overload-induced cardiac hypertrophy and fibrosis primarily by attenuating the inflammatory response, and the protective effects of GAA may involve NF-κB signaling.

## 4. Discussion

Cardiac hypertrophy serves as a major pathological mechanism underlying the progression of diverse cardiovascular diseases. Currently, pharmacological interventions capable of reversing maladaptive cardiac remodeling and restoring function remain limited, highlighting the need for novel therapeutic strategies. *Ganoderma lucidum* (*G. lucidum*), a well-established medicinal fungus in traditional Chinese medicine, has been extensively used in clinical applications. GAA, the principal bioactive compound derived from this species, displays notable chemical stability and a broad spectrum of biological activities [11]. In the present investigation, we showed that GAA significantly alleviates cardiac hypertrophy resulting from chronic pressure overload. Notably, treatment with GAA inhibited TAC-induced myocardial hypertrophy and fibrosis, and enhanced cardiac performance. Further studies revealed that GAA alleviates pressure overload-induced cardiac hypertrophy and fibrosis primarily by attenuating the inflammatory response, and the protective effects of GAA may involve NF-κB signaling.

Cardiac hypertrophy is defined by enlargement of cardiomyocytes, thickening of ventricular walls, and dysregulated expression of genes related to hypertrophic remodeling. Chronic low-grade inflammation involves elevated inflammatory cytokine levels under physiological or environmental stress, resulting in persistent, systemic immune activation. Substantial clinical and experimental evidence has established chronic inflammation as a significant risk factor for cardiovascular diseases [8]. Immune cell infiltration and cytokine production are closely linked to hypertension and subsequent end-organ injury. In essential hypertension, sustained pressure overload drives the recruitment of inflammatory cells and pro-inflammatory cytokines, which contribute critically to myocardial hypertrophy and remodeling [9]. NF-κB is a multifunctional transcription factor critically involved in regulating immune and inflammatory responses, and has been closely associated with a range of cardiovascular conditions such as myocardial infarction, ischemia–reperfusion injury, pathological cardiac hypertrophy, and heart failure [29,30]. Angiotensin II stimulates NF-κB activation in cardiac fibroblasts, cardiomyocytes, and vascular smooth muscle cells [31]. Conversely, suppression of NF-κB signaling reduces hypertrophic effects triggered by angiotensin II [32]. Consequently, dual targeting of both NF-κB activation and angiotensin II-mediated hypertrophic pathways may provide a novel treatment strategy for heart failure. In this investigation, GAA attenuated cardiac hypertrophy by attenuating inflammatory responses. The cardio-protective benefits of GAA were abolished by co-treatment with the NF-κB agonist CUT129 in both cellular and animal models. These findings suggest that GAA alleviates pressure overload-induced cardiac hypertrophy and fibrosis primarily by attenuating the inflammatory response and the protective effects of GAA may involve NF-κB signaling.

Cardiac fibrosis is a common pathological feature during the development of cardiac hypertrophy and structural remodeling [33,34]. Reducing fibroblast accumulation and activation is considered a crucial strategy for attenuating fibrosis and improving cardiac function [35,36]. In the current study, GAA markedly attenuated cardiac fibrosis, demonstrated by a decrease in collagen deposition and reduced expression of fibrosis-associated genes. Moreover, the anti-fibrotic actions of GAA were negated upon administration of an NF-κB agonist in mice subjected to TAC. These findings suggest that GAA may hold therapeutic potential for mitigating fibrosis associated with cardiac hypertrophy.

In our previous study, we investigated another compound from the fruiting body *Ganoderma lucidum* polysaccharides (GLPs), and we demonstrated that GLPs inhibit cardiac hypertrophy through downregulating key genes for hypertrophy and fibrosis, and attenuate pressure overload-induced pathological cardiac hypertrophy by activating PPARγ [37]. This study further expands the application of *Ganoderma lucidum* as a traditional Chinese medicine in the field of cardiac hypertrophy. GAA is present at relatively high levels in the fruiting bodies of *Ganoderma lucidum* and is one of the most abundant triterpenoids in this fungus, making it a potential marker component for evaluating the quality of *Ganoderma lucidum* [38]. Additionally, research on the metabolites of GAA and their pharmacokinetics has been relatively well established [11]. Compared with GLPs, GAA demonstrates advantages including being present in higher levels in *Ganoderma lucidum* fruiting bodies, well-characterized in vivo metabolism and pharmacokinetics, and widespread recognition in the field. This study further expands the application of *Ganoderma lucidum* as a traditional Chinese medicine in the field of cardiac hypertrophy.

This study has several limitations. Other parallel hypertrophic signaling pathways, such as calcineurin–NFAT, MAPK, and PI3K–Akt, were not examined. The molecular mechanisms by which GAA regulates cardiac hypertrophy still warrant further investigation. Additional studies are needed to elucidate the precise pathways through which GAA confers protection against pathological cardiac hypertrophy.

## 5. Conclusions

In summary, our results demonstrate that GAA alleviates pressure overload-induced cardiac hypertrophy and fibrosis primarily by attenuating the inflammatory response, and the protective effects of GAA may involve NF-κB signaling. These findings offer valuable mechanistic insights supporting the potential use of GAA as a therapeutic agent for pathological cardiac hypertrophy and heart failure.

## Data Availability

The original contributions presented in this study are included in the article/Supplementary Materials. Further inquiries can be directed to the corresponding author.

## Supplementary Materials

The following supporting information can be downloaded at: https://www.mdpi.com/article/10.3390/cimb48050471/s1.

## Abbreviations

Ang II: angiotensin II; ANP: atrial natriuretic peptide; BNP: B-type natriuretic peptide; Ctrl: control; Col1a1: collagen 1a1; Col3a1: collagen 3a1; EF: ejection fraction; FS: fractional shortening; GAA: ganoderic acid A; H&E: hematoxylin and eosin; HW/BW: heart weight/body weight; IκBα: inhibitor of NF-κB-α; LVID;d: end-diastolic left ventricular internal dimension; LVID;s: end-systolic left ventricular internal dimension; NRVM: neonatal rat ventricular myocyte; NF-κB: nuclear factor-kappa B; PBS: phosphate-buffered saline; qRT-PCR: quantitative real time Polymerase Chain Reaction; TAC: transverse aortic constriction; WT: wild-type; α-SMA: α-smooth muscle actin; β-MHC: beta-myosin heavy chain.
