# Supplementary material for: Ganoderic Acid A Attenuates Pathological Cardiac Hypertrophy by Attenuating Inflammatory Responses
Source: Curr Issues Mol Biol. 2026 May 1;48(5):471. doi: 10.3390/cimb48050471 (PMC13204186; doi:10.3390/cimb48050471)
Supplement: Supplementary file 1 [file cimb-48-00471-s001.zip › table S1.pdf]

Table S1. Sequences of Primers Used in Quantitative Real-Time Polymerase Chain Reaction.

Gene Name	Forward primer	Reverse primer
Anp(rat)	5'-GAGAAGATGCCGGTAGAAGATG-3'	R:5'-ACTTAGCTCCCTCTCTGAGG-3'
Bnp(rat)	5'-CTGCTGGAGCTGATAAGAGAAA-3'	5'-GCGCTGTCTTGAGACCTAA-3'
β-MHC(rat)	F:5'-CCAACACCAACCTATCCAA-3'	R:5'-GCCAATGTCACGGCTCTT-3'
IL-6(rat)	F:5'-GAAGTTAGAGTCACAGAAGGAGTG-3'	R:5'-CTATGAGGTCTACTCGGCAAAC-3'
TNF-α(rat)	F:5'-ACCTTATCTACTCCCAGGTTCT-3'	R:5'-GGCTGACTTTCTCCTGGTATG-3'
MCP-1(rat)	F:5'-GTCTCAGCCAGATGCAGTTAAT-3'	R:5'-AACTACAAGAGAATCACCAGCAG-3'
iNOS(rat)	F:5'-TGGAGCGAGTTGTGGATTG-3'	R:5'-CTACTGGGTCAAAGACAAGAGG-3'
Gapdh(rat)	F:5’-CATCTCCCTCACAATTCCATCC-3'	R:5'-GAGGGTGCAGCGAACTTTAT-3'
Anp(mouse)	F:5'-TCCGATAGATCTGCCCTCTT-3'	R:5'-CTCCAATCCTGTCAATCCTACC-3'
Bnp(mouse)	F:5'-ACCACCTTTGAAGTGATCCTATT-3'	R:5'-GCAAGTTTGTGCTCCAAGATAAG-3'
β-MHC(mouse)	F:5'-CCGAGTCCCAGGTCAACAA-3'	R:5'-CTTCACGGGCACCCTTGGA-3'
Gapdh(mouse)	F:5'-GTGGCAAAGTGGAGATTGTTG-3'	R:5'-CGTTGAATTTGCCGTGAGTG-3'
α-SMA(mouse)	F:5'-AGGGAGTGATGGTTGGAATG-3'	R:5'-GGTGATGATGCCGTGTTCTA-3'
Col1a1(mouse)	F:5'-AGGCTTCAGTGGTTTGGATG-3'	R:5'-CACCAACAGCACCATCGTTA-3'
Col3a1(mouse)	F:5'-CCCAACCCAGAGATCCCATT-3'	R:5'-GAAGCACAGGAGCAGGTGTAGA-3'
Fibronectin(mouse)	F:5'-CCGGTGGCTGTCAGTCAGA-3'	R:5'-CCGTTCCCACTGCTGATTTATC-3'
IL-6(mouse)	F:5'-CCAGAGCTGTGCAGATGAGT-3'	R:5'-AGCTGCGCAGAATGAGATGA-3'
TNF-α(mouse)	F:5'-AACCTCCTCTCTGCCATCAA-3'	R:5'-CCAAAGTAGACCTGCCCAGA-3'
MCP-1(mouse)	F:5'-ACCTCAGAAACCCTGAGAGA-3'	CTATAACTTCCAGGTCTGTGTGG-3'

iNOS(mouse)	F:5'-GGAATCTTGGAGCGAGTTGT-3'	R:5'-CTACTGGGTCAAAGACAAGAGG-3'

ANP: Atrial natriuretic peptide; BNP: b-type natriuretic peptide; β-MHC: β-myosin heavy chain; Col1a1: collagen I; Col3a1: collagen III; α-SMA: α-smooth muscle actin.
